# Interventions for promoting and optimizing breastfeeding practices: An overview of systematic review

**DOI:** 10.3389/fpubh.2023.984876

**Published:** 2023-01-24

**Authors:** Mahalaqua Nazli Khatib, Abhay Gaidhane, Shilpa Upadhyay, Shital Telrandhe, Deepak Saxena, Padam Prasad Simkhada, Shailendra Sawleshwarkar, Syed Zahiruddin Quazi

**Affiliations:** ^1^Global Evidence Synthesis Initiative, Division of Evidence Synthesis, Jawaharlal Nehru Medical College, Datta Meghe Institute of Higher Education and Research, Wardha, Maharashtra, India; ^2^Centre of One Health, School of Epidemiology and Public Health, Jawaharlal Nehru Medical College, Datta Meghe Institute of Higher Education and Research, Wardha, Maharashtra, India; ^3^Department of Research and Development, Datta Meghe Institute of Higher Education and Research, Wardha, Maharashtra, India; ^4^i Health Consortium, Department of Epidemiology, Indian Institute of Public Health, Gandhinagar, Gujarat, India; ^5^Global Consortium of Public Health Research, School of Human and Health Sciences, University of Huddersfield, Huddersfield, United Kingdom; ^6^Postgraduate Coursework Programs, Faculty of Medicine and Health, Sydney Medical School, The University of Sydney Institute for Infectious Diseases (Sydney ID), University of Sydney, Camperdown, NSW, Australia; ^7^South Asia Infant Feeding Research Network (SAIFRN), Jawaharlal Nehru Medical College, Datta Meghe Institute of Higher Education and Research, Wardha, Maharashtra, India

**Keywords:** promoting, breastfeeding practices, optimizing, LMIC, overview

## Abstract

**Background:**

Optimal breastfeeding (BF) practices are essential for child survival and proper growth and development. The purpose of this overview is to evaluate the effectiveness of different interventions for promoting and optimizing breastfeeding.

**Methods:**

We included systematic reviews (SRs) [including trials from Low-Income (LICs) and Low Middle-Income countries (LMICs)] that have evaluated the effect of various interventions for promoting and optimizing breastfeeding and excluded non-systematic reviews, and SRs based on observational studies. We searched various electronic databases. We followed the standard methodology as suggested by the Cochrane Handbook for Systematic Reviews of Interventions. Two sets of reviewers undertook screening followed by data extraction and assessment of the methodological quality of included SRs.

**Result:**

We identified and screened 1,002 Cochrane SRs and included six SRs in this overview. Included SRs reported only two of the primary outcomes, early initiation of breastfeeding (EIBF) and/or exclusive breastfeeding (EBF). None of the included SR reported continued BF up to 2 years of age. The results were evaluated using two major comparisons groups: BF intervention against routine care and one type of BF intervention vs. other types of BF intervention. Overall results from included SRs showed that there were improvements in the rates of EIBF and EBF among women who received BF intervention such as BF education sessions and support compared to those women who received only standard care. However, BF intervention *via* mobile devices showed no improvements. In Target Client Communication (TCC) *via* mobile devices intervention group, no significant improvements were reported in BF practices, and also the reported evidence was of very low certainty.

**Conclusion:**

Community Based Intervention Packages (CBIP) delivered to pregnant and reproductive-age women during their Antenatal care (ANC) and/or Postnatal care (PNC) periods by Ancillary Nurse-Midwives reported the highest improvement in EIBF compared to women who received standard care. However, insufficient evidence was reported to suggest that BF intervention showed improvements in EBF in both the comparison groups. This overview highlighted the gaps in primary research regarding the uncertainty about the settings such as LICs or LMICs, lack of evidence from LMICs, and also identified gaps in the availability of reliable up-to-date SRs on the effects of several BF interventions to promote and optimize practices.

**Systematic review registration:**

https://www.crd.york.ac.uk/prospero/display_record.php?ID=CRD42020174998, PROSPERO [CRD42020174998].

## Background

### Description of the condition

Optimal breastfeeding practices which include early initiation of breastfeeding (EIBF) within 1 h of birth, exclusive breastfeeding (EBF) for the first 6 months of age, and continued breastfeeding (CBF) for 2 years of age or beyond with complementary foods are vital for child survival and proper growth and development.

Globally, due to under-nutrition, more than 50% of child mortality has been ascribed to insufficient breastfeeding and/or complementary feeding (1). Children with inappropriate breastfeeding are more prone to develop infections such as respiratory infections (2, 3), gastroenteritis (2–4), and otitis media (3, 5), leading to increased hospitalization (6), morbidity, and mortality (7–10). Inadequately breastfed children are at elevated risk of juvenile diabetes and obesity (11) and have compromised intelligence (11–14), educational (15), behavioral (16), and neurodevelopmental outcomes (8). Evidence also indicates that women who inappropriate breastfeed their babies are more prone to develop breast cancer, ovarian cancer, osteoporosis, and diabetes (3, 17–19).

Breastfeeding provides an economic advantage to society in addition to short- and long-term maternal and child health benefits (1, 20, 21). If 90% of US families abide by exclusively breastfeeding for 6 months, the United States would save 13 billion USD per year and avert surplus 911 mostly infants deaths (22). Investing in interventions to promote EIBF, EBF and longer BF durations may be cost-effective (22).

In the year 2003, the World Health Organization (WHO) recommended that babies should be exclusively breastfed throughout the initial 6 months of age and continued for at least 2 years of age (23). According to the CDC's 2018 Breastfeeding Report Card, fewer than half of newborns in the United States were exclusively breastfed for 3 months, and around a quarter were exclusively breastfed for 6 months (24). In many other high-income countries, initiation rates continued to be slightly low, particularly in low-income groups (24). A survey data from the South-Asian countries showed that EIBF and EBF escalated in India, Nepal, and Bangladesh from 1990 to 2016 (25). CBF remained fairly constant across South Asia (25). Improvement in optimal breastfeeding practices is of particular concern in Pakistan and Afghanistan (25). This data indicates that mothers may not be receiving the needed breastfeeding support from family members, health care providers, and employers.

### Description of the interventions

There is currently a range of different interventions for supporting breastfeeding that may target pregnant women, their spouses, family members, the health service, or wider communities.

Telephone-based peer support intervention comprises health education imparted through mobile or telephones to women in ante-natal or post-natal periods. It is effective in initiating and maintaining breastfeeding and improving satisfaction with feeding (26, 27). Interventions aimed to a woman alone or her family members comprise health education imparted through skills training, mother-baby contact, and peer support and that may be offered to one-on-one or groups, in formal or informal settings and be delivered by maternity support workers, health professionals, peers, or social media (28). Peer support interventions that support and promote breastfeeding entails communication between a pregnant woman and a woman with breastfeeding experience from the same background (29). This form of mother-to-mother assistance has been found to boost initiation, exclusivity, and/or continuation rates of BF (27, 30, 31). Peer supporters can undertake training, can be paid or unpaid can be separated or incorporated into the healthcare team (32). Mass or social media campaigns are initiatives directed at the general public that, when combined with other interventions, show some success in encouraging breastfeeding (33).

WHO/United Nations International Children's Emergency Fund (UNICEF), Baby-Friendly Hospital Initiative (BFHI) also referred to as the Baby-Friendly Initiative (BFI) has been proven to be the most effective health care intervention for promoting breastfeeding initiation. The BFHI/BFI is a comprehensive, systematic program that includes organizational change (34, 35). It includes execution of the Ten Steps to effective breastfeeding which involve staff training policies, breastfeeding promotion, and support, infant formula, restricting the use of teats and pacifiers, and keeping mothers and infants together (36). The WHO/UNICEF Baby-friendly Hospital Initiative has been shown to improve breastfeeding rates (37–39) however ambiguity remains regarding effective approaches to improve BF in community health care services (40, 41). According to reviews, interventions such as counseling and health education offered by healthcare and non-healthcare professionals as well as peer support have escalated the percentage of women who early initiate BF, exclusively breastfeed, and continue to breastfeed for a longer duration (26, 32, 33, 35, 42, 43).

### How the interventions might work

Breast milk is regarded as the best and only source of nutrition for all newborns from the time of birth to the age of 6 months. The nutritional benefits are due to potent immune boosters and a specialized composition that meets babies' proper growth and development requirements (13). Breast milk includes growth factors, hormones, cytokines, cells, etc., and offers several benefits over cow's milk or soy protein infant formulae (19). In the health setting, peer support is a “created” social interaction that aims to improve health care delivery (26). Peer volunteers are trained to offer “emotional, informational, and appraisal” support to improve breastfeeding outcomes by boosting wellbeing and social connectivity. By using their experiential expertise and training, the peers may provide a number of solutions and guidance on parenting and feeding challenges confronting new mothers (27). Volunteer training emphasizes the importance of assisting the mother in making her own decisions and referring the mother to professional help when necessary (27).

### Why it is important to do this overview

The key step to meet WHO recommendations for breastfeeding is the escalating rates of early initiation of breastfeeding and realizing the probable role of breastfeeding in health improvement, minimizing the economic burden of illness, and minimizing health disparities. A variety of individual studies and systematic reviews have assessed a comprehensive range of support interventions for breastfeeding. However, we still need to learn more about what works best to support breastfeeding. There is a need to evaluate all potentially associated BF interventions for systematically promoting breastfeeding practices.

To the best of our knowledge, no published overview has assembled and summarized the evidence from systematic reviews on breastfeeding interventions, to aid health professionals, consumers, researchers, funding bodies, policymakers/guideline developers in decision-making and evidence translation. The aim of this overview is to assess interventions for promoting women to breastfeed, to assess their efficacy in terms of changes in the percentage of women who early initiate breastfeeding, who breastfeed exclusively, and who continue to breastfeed their children up to 2 years of age. We will identify existing knowledge gaps and can provide clear suggestions and recommendations for future systematic reviews and clinical research.

## Objectives

### Primary objective

The objective of this overview is to summarize the evidence from systematic reviews on the impact of different interventions designed to promote and optimize early initiation of breastfeeding (EIBF), exclusive breastfeeding (EBF) for the first 6 months of life and continued breastfeeding up to 2 years of age and to assess the effects of these interventions on associated outcomes, including infant mortality.

### Secondary objectives

1. To describe different types of breastfeeding support (evaluated in systematic reviews) in terms of the timing and intensity of interventions and the settings (differential impact on different subgroups of the population) in which they have been used.
2. To assess whether interventions delivered in both antenatal (ANC) and postnatal periods (PNC) are more effective than those delivered only in the postnatal period.
3. To compare the efficiency of different care providers (who had given interventions).
4. To explore appropriate strategy for supporting women who desire early initiation of breastfeeding, exclusive breastfeeding for the first 6 months of life, and continued breastfeeding up to 2 years of age.

## Methods

### Criteria for considering reviews for inclusion

#### Types of studies

We included only Cochrane systematic reviews (that had included randomized clinical trials including cluster or quasi-randomized trials) evaluating the effect of various interventions for promoting and optimizing EIBF, EBF for the first 6 months of life, and continued breastfeeding up to 2 years of age. We excluded non-Cochrane SRs, non-systematic reviews, and SRs based on observational studies. We included the updated SRs. There were no restrictions on the language or publication status of systematic reviews.

#### Types of participants

Participants included were pregnant women, women who were breastfeeding their babies, and women who are willing to breastfeed in the future. We included SRs including trials from LICs and LMICs as defined by World Bank (based on Atlas Gross national per capita estimates). We excluded SRs based on trials from HIC and UMICs. However, we included SRs that had participants irrespective of countries as defined by the World Bank (LICs, LMICs, HICs, UMICs), but reported separate data for LICs and LMICs as subgroup analysis. We imposed no restriction on race/ethnicity, and the type of settings from where the participants were recruited. SRs focussed specifically on women and children with additional care needs or a specific health problem, e.g., mothers with diabetes, HIV/AIDS or infants with cleft palate, or premature babies, were excluded from this overview.

#### Types of interventions

“Support” interventions eligible for this overview may range from m-health, Behaviour Change Communication (BCC), health education, health systems and policy interventions like Health Sector Initiatives (HIS), Infant Young Childhood Feeding (IYCF), specialized clinics, workplace interventions, positive parenting interventions, and/or combination of interventions. We included SRs in which the intervention occurred only in the postnatal period (PNC) or in conjunction with an antenatal (ANC) component. Intervention could be offered by health professionals laypeople, or peers, in either hospital or community settings in LICs and LMICs.

#### Types of comparisons

We included SRs that have compared breastfeeding support intervention vs. routine care or one form of intervention vs. the other.

#### Types of outcomes

1. Primary outcomes

- Early initiation of breastfeeding.
- Exclusive breastfeeding for the first 6 months of life.
- Continued breastfeeding up to 2 years of age.

2. Secondary outcomes

- Acceptability: Any measure of acceptability.
- Satisfaction: Any measure of satisfaction.

### Search methods for identification of reviews

We searched the Cochrane Database for Systematic Reviews (CDSR) for identifying Cochrane reviews and additional databases like PubMed, DARE, CINAHL, PsychINFO, Google Scholar for non-Cochrane Reviews if deemed necessary. We searched the Cochrane Pregnancy and Childbirth Editorial Base and Cochrane Child Health Editorial Base to seek any relevant reviews or review updates in progress, and The International Prospective Register of Systematic Reviews (PROSPERO) for SR protocols at https://www.crd.york.ac.uk/prospero/. We contacted the Protocol authors for a pre-publication version of SRs. Additionally; we searched the reference lists of retrieved studies. We used medical subject heading and text word terms and tailor the search to individual databases. We used keywords and synonyms to sensitize the search. We searched all databases from their creation to the present and we did not restrict the language of publication status.

The protocol of this overview of systematic review was registered in PROSPERO (International prospective registration of systematic reviews) https://www.crd.york.ac.uk/prospero/display_record.php?ID=CRD42020174998.

The registration number of the proposed protocol is CRD42020174998. We conducted our search on April 28, 2021 and update it on November 13, 2021, to include newly added SRs.

The search strategy for CENTRAL was as:

Search Strategy for CENTRAL *via* Cochrane library

ID Search

#1 “breast feed^*^”

#2 “breast fed”

#3 “breastfeed^*^”

#4 “breast-fed”

#5 MeSH descriptor: [Breast Feeding] explode all trees

#6 #1 Or #2 OR #3 OR #4 OR #5.

### Data collection and analysis

We followed the standard methodology as suggested by Part 1: Chapter V of Cochrane Handbook for Systematic Reviews of Interventions (44).

### Selection of reviews

Two reviewers initially screened the titles and abstracts of all the included SRs using the “Rayyan” software (45) and all seemingly eligible SRs were moved for the next step screening on full texts. A PRISMA diagram (46) was prepared to keep track of the search process. Disagreements amongst the primary reviewers were resolved by discussion or consultation with a third reviewer. We presented a reason for exclusion where relevant.

### Data extraction and management

One reviewer had extracted data from included systematic reviews into a Microsoft Excel file using a pre-designed data extraction form, which was pilot-tested for its suitability and usability. A second reviewer had checked the data extraction. The discrepancies were resolved through discussion or consultation with a third reviewer. We had created “Characteristics of Reviews” tables for both included and excluded reviews including the following information:

- Basic review information (review title, author, last assessed as up-to-date).
- Study information (number of included trials, the sample size in range).
- Population (settings, age, demographics, specific definitions of population, inclusion criteria, and other important participant characteristics).
- Interventions (type, frequency, intensity, duration, personnel delivering the intervention).
- Comparison.
- Outcome measures for which data are reported (for each prespecified outcome: Outcomes reported, the definition of the outcome, number of trials and participants for each outcome; time of measurement of outcome, and the reported effect estimate).

Wherever possible, we contacted review authors to clarify data included in systematic reviews or to inquire about missing data. We had attempted to include only one review per intervention to avoid the duplication of evidence and resultant double-counting of trial data, by including only the most current and inclusive systematic review on a given intervention. All the included SRs varied in interventions.

We made decisions transparent in the main text as well as in tables for the overview. Data extraction had driven by well-defined review questions and not by the individual review data. We extracted additional data from original trials for enhancing the quality of the overview. Where the data was not presented in the required format, we re-extracted the data from original SRs by using Review Manager (RevMan).

### Assessment of methodological quality of included reviews

#### Quality of included reviews

One reviewer assessed the methodological quality of included SRs with the Revised Assessment of Multiple Systematic Reviews (R-AMSTAR) tool (47), and a second reviewer checked these assessments for accuracy. Any disagreements amongst the primary reviewers were resolved by discussion or consultation with a third reviewer.

The R-AMSTAR tool consisted of the following questions to be answered with either “yes,” “no,” or “unclear.” One point was awarded for every question answered “yes” for the highest possible score of 11. High-quality reviews score 8 or higher; moderate-quality score 4–7; low-quality systematic reviews score 3 or fewer “yes” answers.

1. Was an “a priori” design provided?
2. Was there duplicate study selection and data extraction?
3. Was a comprehensive literature search performed?
4. Was the status of publication (i.e., gray literature) used as an inclusion criterion?
5. Was a list of studies (included and excluded) provided?
6. Were the characteristics of the included studies provided?
7. Was the scientific quality of the included studies assessed and documented?
8. Was the scientific quality of the included studies used appropriately in formulating conclusions?
9. Were the methods used to combine the findings of studies appropriate?
10. Was the likelihood of publication bias assessed?
11. Was the conflict of interest stated?

We excluded systematic reviews that did not meet the minimum quality standards of a rating of at least 4. We recorded the R-AMSTAR assessments as a tables to the overview.

### Quality of included studies within reviews

We reported and not reassessed the “Risk of bias” of included trials in systematic reviews as given by authors of SRs.

#### Quality of evidence in included reviews

We reported the quality of evidence of relevant outcomes (GRADE assessments) as presented in the “Summary of findings” tables of the included SRs; if provided. We did not exclude evidence of “low” or “very low” quality.

#### Data synthesis

We organized the evidence in texts and tables according to each of our pre-specified outcomes, rather than by intervention. We concentrated primarily on describing and classifying the intervention and its efficacy. We created a data synthesis table for each review that included the following outcomes: characteristics of included systematic reviews, summary effect estimates, and the GRADE assessments. Toward the end of data extraction, we further chalked out the process of structure, categorization, and analysis of tables. One reviewer had created data synthesis tables and another overview author checked the tables for accuracy. We had resolved the discrepancies by discussion and consultation with another reviewer. We compared the pooled estimates of effectiveness for each intervention to the comparator in SRs that contained meta-analyses. We presented the data by the outcome and not by intervention in texts and tables. If data permits, we had chosen to use a fixed-effects model when the data was not heterogeneous or use the random effects model in presence of heterogeneity. We tried to present the data as risk ratio for dichotomous outcomes and Mean Difference (MD) for continuous outcomes. Where the data was not presented in the required format, we re-extracted the data from original SRs by using Review Manager (RevMan). Where the systematic reviews did not include meta-analyses; we incorporated brief narrative assessments.

“Effectiveness statement”, We combined the GRADE assessment with the R-AMSTAR rating in order to summarize all intervention decisions in a single, concise judgement (48).

### Analysis of subgroups or subsets

Based on the availability of data, we made appropriate categorizations of results in terms of the region (rural/urban), population subgroup, and type of intervention.

### Summarizing the results

We summarized the results based on the outcome, type of interventions, and quality of evidence.

## Results

### Search results

**Figure 1:**
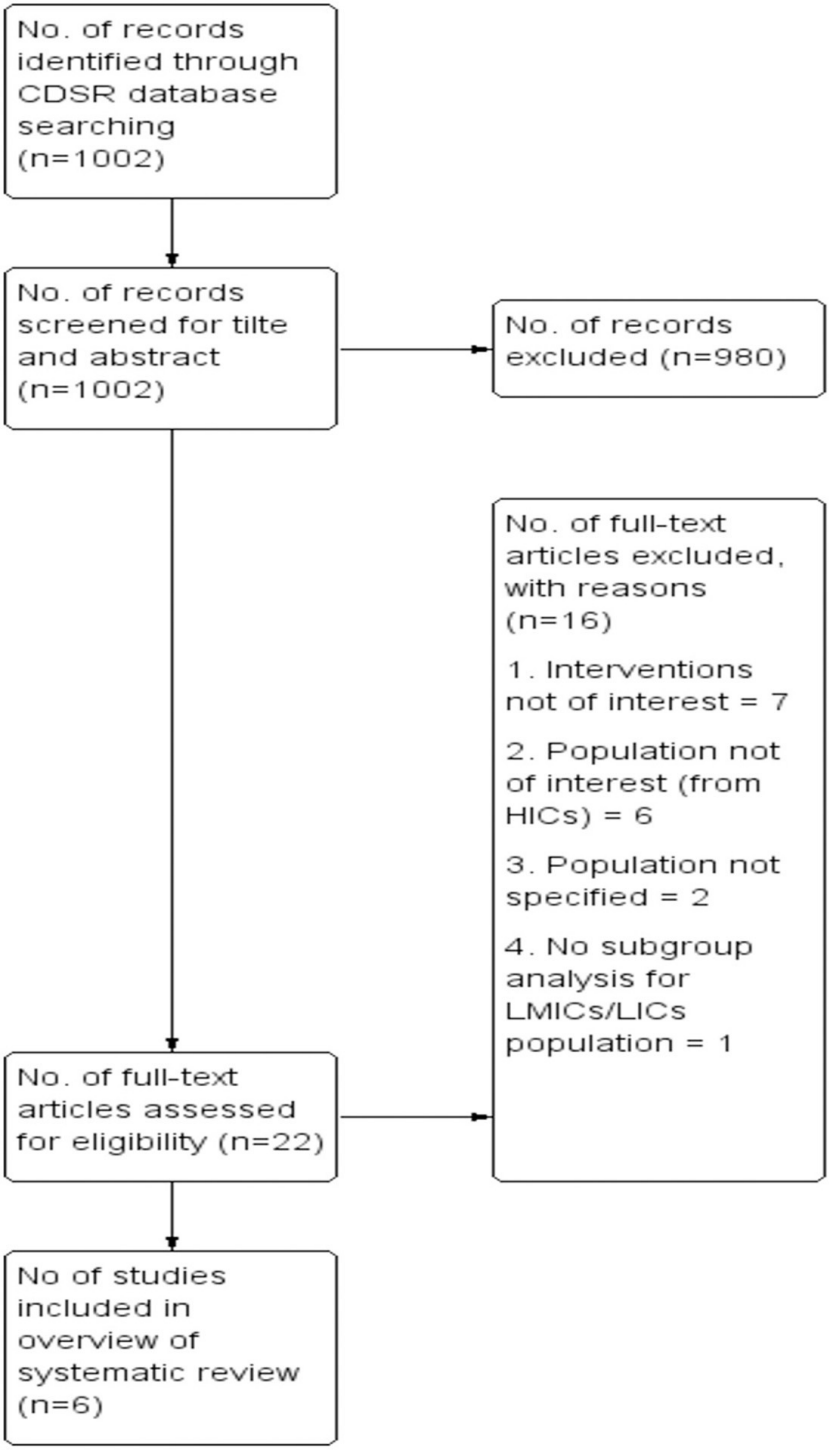
PRISMA flow diagram.

We identified and screened a total of 1,002 records from the Cochrane Database of Systematic Reviews (CDSR), written in the English language on interventions for pregnant women or women of reproductive age to promote and optimize breastfeeding practices. We excluded 980 systematic reviews (SRs) after the screening of title and abstract and screened 22 SRs full-texts for eligibility. Then, after excluding 16 SRs, we included six SRs that matched our inclusion criterion. Three of the six included SRs (43, 49, 50) were the updates of earlier reviews (51–53). We documented the selection process in the form of a PRISMA Flow diagram (Figure 1).

### Description of included studies

#### Details of included systematic reviews

We examined six SRs (42, 43, 49, 50, 54, 55) from the Cochrane database. Included SRs were aimed to promote BF practices such as EIBF, EBF, and continued BF for up to 2 years, etc. All the included SRs followed randomized controlled trials (RCTs), cluster-randomized trials, or quasi-randomized trials as their research design. Except for one SR (49), all five SRs were published on and after 2015, Two of the six SRs (50, 54) included participants exclusively from LMIC or LIC, whereas the other three SRs (42, 43, 55) included participants from a range of low to high-income countries, although we extracted the data solely from LIC/LMIC. Of the six included SRs, one SR (49) addressed interventions delivered in the workplace to encourage BF practices although there was no study reported related to interventions delivered at the workplace to support BF. We have presented a comparison of the characteristics of the included studies in Table 1.

**Table 1:** Characteristics of included studies.

**References**	**Last assessed as up-to-date**	**No. of trials/no. of participants**	**Inclusion criteria for study design**	**Inclusion criteria for population**	**Inclusion criteria for intervention**	**Comparison**	**Outcomes**	**Conclusion**
Lumbiganon et al. (43)	1st March 2016	No. of trials: 24 (two studies from LMIC) **Participants:** Total: 390 IG: 196 CG: 194	Cluster RCT	Women attending monthly microcredit meetings	**Interventions**: Monthly BF education + weekly cell phone messages **Content**: Multiple methods of BF education (weekly cell phone BF messages and monthly face-to-face BF information)	Standard care	1. EIBF 2. EBF at 3 and 6 months	Insufficient evidence to support that any type of BF education session vs. SC improved EIBF.
Lassi et al. (54)	2nd May 2017	No. of trials: 33 **Participants**: 1,26,375	1. Community-based-RCT 2. Cluster-RCT 3. Quasi-RCT	Pregnant women, mothers of neonates, women of reproductive age, caregivers	**Interventions**: Any combination of CHEI and any ANC **Delivered by**: HCP or CHW **Content**: CHEI includes 1. Promotion of routine ANC 2. Maternal health education 3. Promotion of EIBF and EBF 4. KMC 5. Newborn resuscitation 6. Management of neonatal infections **Mode of delivery:** 1. One-to-one counseling 2. Group counseling 3. Mass media	Usual health services	1. EIBF	Any combination of CHEI during any period (ANC/PNC) given to mothers or family members or both were found to improve BF practices.
Palmer et al. (55)	July 2019	No. of trials: 11 (four from LMICs and 1 from LIC) **Participants** 5,497 (from LMICs and LIC)	RCTs	Pregnant and postpartum mother's and caregivers	**Interventions**: TCC *via* mobile devices **Delivered by:** HCW **Content**: Delivered TCC *via* mobile devices to improve maternal, new-born or child health or a combination of both	1.Standard care 2. TCC *via* non-digital communication 3. Digital non-targeted communication	1. EBF	No improvement was reported in BF practices as 100% of women were involved in exclusive breastfeeding
Balogun et al. (42)	29th February 2016	No. of trials: 28 (three from LMICs and 1 from LIC) **Participants**: Nicaragua: unclear Malawi: 55,931 Nigeria: 461 Ghana: NR	RCTs, Cluster-RCTs	Pregnant and reproductive age group (1, 15–47) group women from LIC (Malawi) and LMIC (Nicaragua, Nigeria, and Ghana)	**Interventions**: BF education and support and Early mother-infant contact **Delivered by:** Non-HCPs (women's group, peer counselors) **Content**: Education and support on BF education.	Standard care	1. EIBF	In LIC, BF interventions provided by non-HCPs had reported improvements in EIBF rates
Lassi and Bhutta (50)	25 May 2014	No. of trials: 26 **Participants**: Total: 72.464 IG: 37,813 CG: 34,651	Community-based RCT, cluster RCT, quasi-RCT	Pregnant and reproductive age group women from developing countries	**Interventions**: CBIP **Delivered by**: ANMs **Content**: CBIP including additional training of LHW/visitor, community midwives, CHW/VHW, facilitators, or TBAs in maternal care.	Usual maternal and newborn care services	1. EIBF within 1 h of birth	CBIP were found to improve BF practices
Abdulwadud and Snow (49)	2 August 2012	No included studies and no participants were recruited	RCTs, Cluster or quasi RCTs	Women in full-time or part-time employment in both private and public sectors return to paid work after giving birth.	**Interventions**: interventions to support BF at the workplace **Content**: Any type of workplace strategy, to encourage, assist and support BF practices for women returning to work after giving birth.	No intervention or two or more workplace interventions against each other.	No outcomes reported	No conclusion can be drawn

LMIC, low middle income country; LIC, low income country; IG, intervention group; CG, control group; RCTs, randomized controlled trials; ANC, antenatal care; PNC, postnatal care; LC, lactation consultation; SC, standard care; BF, breastfeeding; EIBF, early initiation of breastfeeding; EBF, exclusive breastfeeding; CHEI, community health educational intervention; HCP, health care professionals; CHW, community health workers; VHW, village health workers; KMC, kangaroo mother care; HCW, health care workers; TCC, targeted client communication; HBC, health behavior change; CBIP, community based intervention packages; ANM, ancillary nurse-midwives; LHW, lady health workers; TBA, traditional birth attendants; NR, not reported.

### Details of participants in included SRs

Six of the included SRs contributed data for analysis and reported interventions to promote breastfeeding practices. Lumbiganon et al. (43), conducted an SR on women attending monthly microcredit meetings. Pregnant women, mothers of neonates, women in their reproductive age, mothers in their postpartum period, partners/spouses, or family members exclusively or mostly from LMICs or LICs were all the recipients of the BF interventions in four included SRs (42, 50, 54, 55). In addition, Abdulwadud et al. (49) performed an SR that targeted women in their full-time or part-time employment in both the commercial and public sectors who were returning to paid work following maternity break. Two of the six SRs (50, 54) included participants mainly from LMIC or LIC, the other three SRs (42, 43, 55) included participants from a range of low to high-income countries although we retrieved the data solely from LIC/LMIC. Whereas, one of the SR (49) did not include any study. The number of participants included in the SRs ranged from 390 to 1,26,375. In the included SRs, participants were recruited from antenatal and/or postnatal phases. Four of the included SRs acknowledged the duration of the intervention (42, 50, 54, 55). The other two SRs, on the other hand, did not specify the duration (49, 51).

#### Details of interventions in included SRs

All SRs included in this overview had interventions aimed at optimizing and promoting BF practices, five SRs (42, 43, 50, 54, 55) evaluated the effect of educational interventions, and one SR (49) addressed support interventions for BF practices (including physical facilities, lactation breaks, creches, and nurseries). BF Interventions in the included SRs were delivered by personnel from government, non-governmental, and private organizations (54), health system or health workers (55), non-healthcare professionals (women's group peer counselors) (42), and female ancillary nurse midwives (ANMs) (50). Two of the included SRs (43, 49) did not provide any information regarding the personnel who delivered the BF interventions.

Lumbiganon et al. (43) conducted a review on BF interventions which included weekly cell phone BF text and voice messages to cell phone and monthly face-to-face BF information delivered to women attending monthly microcredit meetings (43). Another review conducted by Lassi et al. (54), focussed on Community Health Education Interventions (CHEI) based on maternal and child health such as group counseling, one-to-one counseling, mass media (television, radio, cellular messages, brochures, newspaper, banners, etc.) or any combination of the above methods delivered to mothers or family members by the government, non-governmental organizations, and private providers. Palmer et al. (55) conducted an SR in both HICs and LICs settings and addressed Targeted Client Communication (TCC) *via* mobile devices delivered to pregnant women and parents of young children by the health system or health workers. An SR conducted by Balogun et al. (42) addressed any intervention such as BF education and support that promotes BF practices such as EIBF and EBF, delivered to pregnant women and reproductive age group women by women's group peer counselors. Lassi and Bhutta (50) promoted additional training (including lectures supervised hands-on training) of community midwives, lady health workers or visitors, community or village health workers, traditional birth attendants (TBAs), or facilitators in maternal care during pregnancy, delivery, and the postpartum period offered to pregnant and women of reproductive age by Auxillary nurse midwives (ANMs) from local government and non-government organizations. According to Abdulwadud and Snow (49), any form of workplace approach to promote, support, and assist BF practices for women returning to work after maternity leave is considered as workplace intervention. This SR, on the other hand, found no studies on workplace interventions to support BF.

Four of the six included SRs (42, 43, 50, 54) included comparison groups that were either standard or routine care whereas one SR (55) had comparison groups both as standard care and another type of intervention which includes TCC with non-digital communication (face-to-face communication, pamphlets, letters). And one SR (49) had two or more workplace interventions compared against each other or no intervention.

#### Details of outcomes in included SRs

All six included SRs evaluated the impact of BF interventions on BF practices such as EIBF, EBF, and continued BF up to 2 years of age. Included SRs reported only two of the primary outcomes, EIBF and/or EBF in this overview. The third primary outcome i.e., continued BF up to 2 years of age as well as secondary outcomes such as acceptability and satisfaction were not assessed in any of the included SRs. The results were evaluated using two major comparisons groups: BF intervention against routine care and one type of BF intervention vs. other types of BF intervention. Four SRs (42, 43, 50, 54) assessed the effect of interventions on EIBF whereas two SRs (43, 55) evaluated the effect of interventions on EBF in BF intervention vs. routine care comparison group. Only one SR (55) reported EBF in another group i.e., one BF intervention vs. another form of BF intervention comparison group.

We retrieved relevant outcomes (reported as events and population size as well as RR) and categorized them for analysis based on the results mentioned below in Tables 2, 3.

**Table 2:** Findings of included studies.

**Outcome**	**References**	**No. of trials/no. of participants**	**Effect estimate**	**Quality of evidence (GRADE assessment)**	**Definition (if given)**	**Effectiveness statement**
EIBF (early Initiation of breastfeeding)	(43)	**Trials**: 1 cluster-randomized trial (LMIC) **Participants**: 390	**EIBF:** RR = 1.44 (95% CI = 1.06, 1.97) *I*^2^ = 23.3%	High	NR	Insufficient evidence to suggest that any antenatal BF education was found to be more effective than standard care for improving EIBF
	(50)	**Trials**: 11 RCTs **Participants**: 72,464	**EIBF within 1 h of birth:** RR = 1.93 (95% CI = 1.55–2.39) *I*^2^ = 98%	NR	NR	CBIP were found to be significantly effective in improving maternal and neonatal health
	(54)	**Trials**: 19 RCTs **Participants**: 1,26,375	**Timely initiation of BF after CHEI**: 100%RR = 1.56, (95% CI =1.37–1.77) *I*^2^ = 99%	NR	NR	CHEI was found to be significantly effective for improving BF practices when given to mothers and other family members
	(42)	**Trials**: 3 RCTs **Participants**: Total: 2,066 IG: 1,064 CG: 1,002	**EIBF:** RR = 1.7 (95% CI = 0.98–2.95) *I*^2^ = 78%	Low	NR	BF interventions provided by non-healthcare professionals reported improvements in EIBF rates but the result was not statistically significant
EBF (exclusive breast feeding)	(43)	**Trials**: 1 cluster-randomized trial (LMIC) **Participants**: 390	**EBF at 3 months:** RR = 1.21 (95% CI = 0.91–1.61) **EBF at 6 months:** RR = 1.47 (95% CI = 1.06, 2.05)	Moderate	NR	Insufficient evidence to suggest that any antenatal BF education was found to be more effective than standard care for improving EBF at 3 or 6 months
	(55)	**Trials**: 1 RCT **Participants**: 40	**Low-risk setting: EBF up to 3 months:** RR = 0.92 (95% CI = 0.79–1.08)	Low	**Low-risk setting:** All women in the control group exclusively breastfed their babies	The intervention provided no significant improvement in BF practices as 100% of women reported EBF to their babies.
Continue BF for 2 years	None of the SRs reported this outcome.	NR	NR	NR	NR	None of the SRs had assessed these parameters. Therefore, SR needs to be conducted considering these parameters.
Acceptability	None of the SRs reported this outcome.	NR	NR	NR		None of the SRs had assessed these parameters. Therefore, SR needs to be conducted considering these parameters.
Satisfaction	None of the SRs reported this outcome.	NR	NR	NR		None of the SRs had assessed these parameters. Therefore, SR needs to be conducted considering these parameters.

Comparison 1: BF intervention vs. routine care. NR, not reported.

**Table 3:** Findings of included studies.

**Outcome**	**References**	**#Trials/ #participants**	**Effect estimate**	**Quality of evidence (GRADE assessment)**	**Effectiveness statement**
EIBF	None of the SRs reported this outcome.	NR	NR	NR	None of the SRs had assessed these parameters. Therefore, SR needs to be conducted considering these parameters.
EBF	(55)	1 RCT 42 participants	**TCC compared to non-digital TCC: EBF (9 months postpartum):** RR = 0.92 (95% CI = 0.79–1.07)	Low	Insignificant improvements were reported in BF practices in the TCC *via* mobile devices group compared to non-digital TCC as 100% of women exclusively breastfed their babies.
Continue BF for 2 years	None of the SRs reported this outcome.	NR	NR	NR	None of the SRs had assessed these parameters. Therefore, SR needs to be conducted considering these parameters.
Accepatability	None of the SRs reported this outcome.	NR	NR	NR	None of the SRs had assessed these parameters. Therefore, SR needs to be conducted considering these parameters.
Satisfaction	None of the SRs reported this outcome.	NR	NR	NR	None of the SRs had assessed these parameters. Therefore, SR needs to be conducted considering these parameters.

Comparison 2: One BF intervention vs. other intervention. NR, not reported.

### Excluded systematic reviews

We excluded 16 SRs (56–71) from this overview following the screening of full texts articles. The most common reasons for exclusion were unrelated interventions and population. Of the 16 excluded SRs, seven SRs (56–62) did not focus on the interventions intended to promote BF practices, six SRs (63–68) did not target the population of interest, two SRs (69, 70) did not specify the population and one SR had no subgroup analysis for LMICs/LICs population (71). We presented the list of excluded studies in Table 4.

**Table 4:** List of excluded studies with reasons.

**References**	**Excluded review**	**Reason for exclusion**
Kramer and Kakuma (58)	Optimal duration of exclusive breastfeeding	Low birth weight babies Interventions not related to BF
Ndikom et al. (60)	Extra fluids for breastfeeding mothers for increasing milk production	Interventions not related to BF
Bryanton et al. (64)	Postnatal parental education for optimizing infant general health and parental infant relationships	Population from HIC
Fair et al. (65)	Interventions for supporting the initiation and continuation of breastfeeding among women who are overweight or obese	Population from HIC
McFadden et al. (71)	Support for healthy breastfeeding mothers with healthy term babies	No segregated data from HIC and LIC
Lewin et al. (59)	Lay health workers in primary and community health care for maternal and child health and the management of infectious diseases	Interventions not related to BF
Gagnon and Sandall (66)	Individual or group antenatal education for childbirth or parenthood, or both	Population from HIC
Sandall et al. (68)	Midwife-led continuity models vs. other models of care for childbearing women	Population from HIC
Barlow et al. (63)	Individual and group based parenting programmes for improving psychosocial outcomes for teenage parents and their children	Population from HIC
Becker et al. (56)	Methods of milk expression for lactating women	Interventions not related to BF
Opiyo and English (61)	In-service training for health professionals to improve care of seriously ill newborns and children in low-income countries	Interventions not related to BF
Pantoja et al. (62)	Implementation strategies for health systems in low-income countries: an overview of systematic reviews	Intervention not related to BF
Ciapponi et al. (57)	Delivery arrangements for health systems in low-income countries: an overview of systematic reviews	Intervention not related to BF
Jaafar et al. (67)	Effect of restricted pacifier use in breastfeeding term infants for increasing duration of breastfeeding	Population from HIC
Jaafar et al. (69)	Rooming-in for new mother and infant vs. separate care for increasing the duration of breastfeeding	Population not specified
Lee and Thomas (70)	Antenatal breast examination for promoting breastfeeding	Population not specified

### Methodological quality of included reviews

The R-AMSTAR grading system was developed to evaluate the procedures employed in Cochrane reviews. All Cochrane reviews followed a general protocol outlining procedures, five of the six included reviews had a high score, while one included SR (49) had a low score. R-AMSTAR ratings for each Cochrane systematic review—Breastfeeding (BF) interventions—(Table 5).

**Table 5:** Methodological quality of included studies.

**S. no**	**Questions**	**Lumbiganon et al. (43)**	**Lassi et al. (54)**	**Palmer et al. (55)**	**Balogun et al. (42)**	**Lassi and Bhutta (50)**	**Abdulwadud and Snow (49)**
1.	Was an “a priori” design provided?	4	4	4	4	4	4
2.	Was there duplicate study selection and data extraction?	4	4	4 (one author extract and other cross-checked)	4	4	4
3.	Was a comprehensive literature search performed?	4	4	4	4	4	4
4.	Was the status of publication (i.e., gray literature) used as an inclusion criterion?	4	4	4	4	4	4
5.	Was a list of studies (included and excluded) provided?	4	4	4	4	4	1 (no included studies)
6.	Were the characteristics of the included studies provided?	3	4	4	3 (data is not complete and accurate)	4	1 (no included studies)
7.	Was the scientific quality of the included studies assessed and documented?	4	4	4	4	3	1 (no included studies)
8.	Was the scientific quality of the included studies used appropriately in formulating conclusions?	2	2	4	3	4	1 (no included studies)
9.	Were the methods used to combine the findings of studies appropriate?	4	4	4	3	4	1 (no included studies)
10.	Was the likelihood of publication bias assessed?	3	3	3	3	3	1 (no included studies)
11.	Was the conflict of interest included?	3	4	3	3	3	3
	**Overall score (out of 44)**	39	41	42	39	41	25

All six SRs (42, 43, 49, 50, 54, 55) had provided a “a priori” design, and data was retrieved by two authors who independently searched and selected studies. All SRs had mentioned the type of publication (published, unpublished, gray literature). Only one SR (49) did not meet the criteria since there were no included studies. Five of the six included SRs provided the list of included and excluded studies. In presenting characteristics of included studies, three SRs (50, 54, 55) scored four ratings, two SRs (42, 43) scored three ratings and one (49) scored one rating (since this SR did not include any study). Five of the included SRs also evaluated the scientific quality of included studies and described statistical methods used to combine findings of included studies. There were no publication biases and conflicts of interest reported in any of the six SRs. All included SRs scored high ratings except for one (49) as this SR did not include any study. All included five SRs (42, 43, 50, 54, 55) were of high quality.

### Effect of interventions

We summarized the key findings of all six included SRs in Tables 2, 3.

These findings provided a summary of the reported effects as well as the degree of certainty of the evidence for each intervention. We have presented the outcomes for all interventions for which independent data were available in two primary comparison groups

1. Breastfeeding support Intervention vs. standard/routine care
2. One form of intervention vs. other types of intervention

None of the included SRs reported the results for both types of comparisons. Included SRs compared the intervention with standard/routine care or with other interventions.

We organized the overall number of studies and a total number of participants randomized for each included SR. We also presented the number of studies and numbers of women randomized to each comparison group.

### Findings of included SRs

#### Comparison 1: BF intervention vs. routine care

##### Primary outcome

###### EIBF:

**Figure 2:**
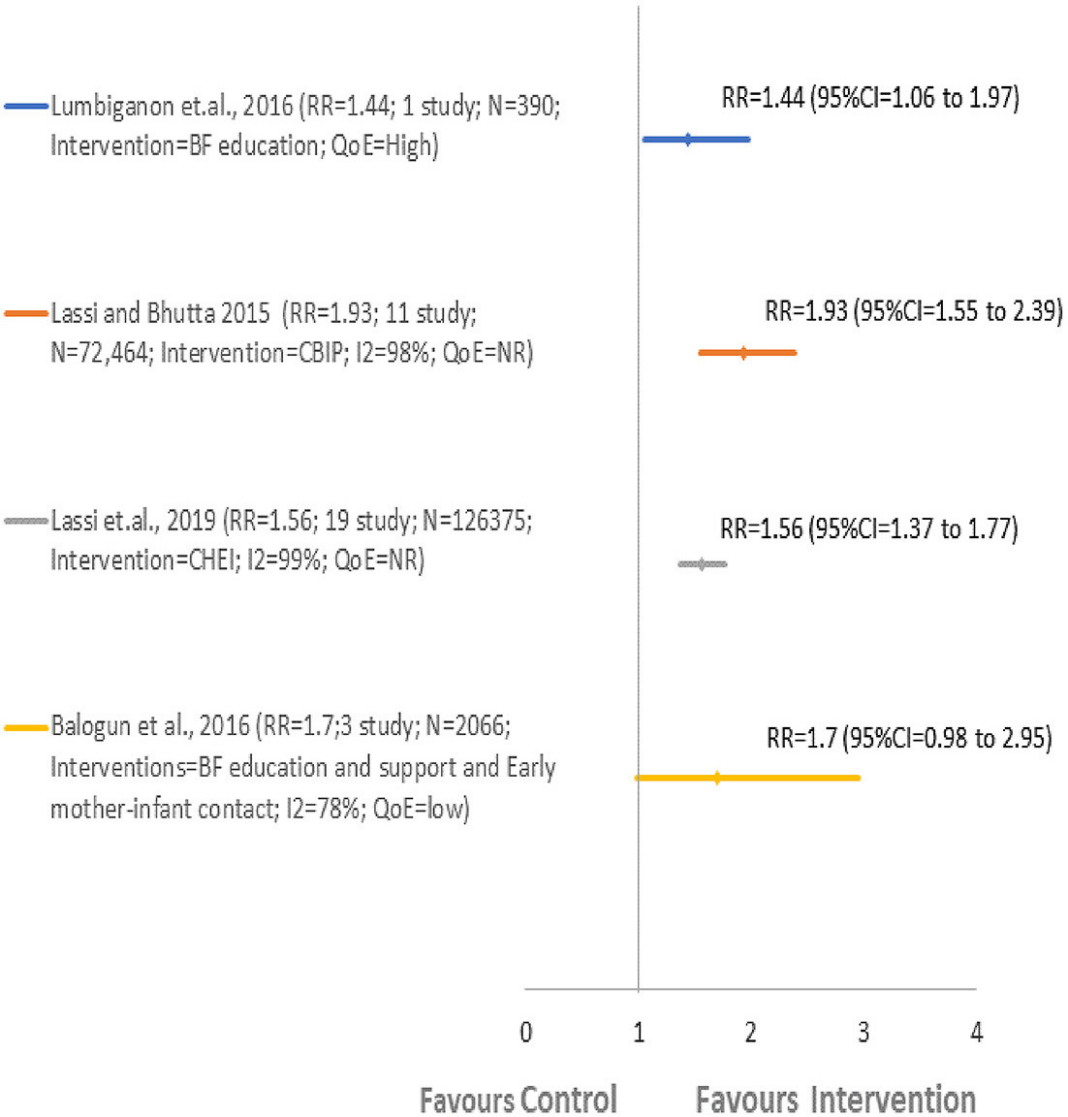
Comparison 1: BF intervention vs. routine care. EIBF, early initiation of breastfeeding.

Four included SRs (42, 43, 50, 54) reported findings on EIBF (Figure 2; Table 2). All the included SRs in this overview agreed that the BF interventions improved EIBF as compared to routine care. The overall improvement in EIBF reported in included SRs varied from 44% (RR = 1.44, 95% CI = 1.06–1.97; one study; 390 participants) (43) to 93% (RR = 1.93, 95% CI = 1.55–2.39; 11 studies; 72,464 participants) (50). Community-Based Intervention Packages delivered to pregnant and reproductive-age women during their ANC and/or PNC period by Ancillary Nurse-Midwives reported the highest improvement in EIBF by 93% (RR = 1.93, 95% CI = 1.55–2.39; 11 studies; 72,464 participants*; QoE* = *NR*) (50), followed by BF education and support and early mother-infant contact administered to pregnant and reproductive age group women during the antenatal period by women's group peer counselor by 70% (RR = 1.7, 95% CI = 0.98–2.95; three studies; 2,066 participants; *QoE* = *low*) (42). Community Health Educational Intervention (CHEI) delivered to pregnant women and mothers throughout both the antenatal and postnatal period by health care workers improved EIBF by 56% (RR = 1.56, 95% CI = 1.37–1.77; 19 studies; 1,26,375 participants; *QoE* = *NR*) (54). Another included SR addressed BF intervention which includes monthly BF education sessions and weekly cell phone messages delivered to women attending monthly microcredit meetings and reported an improvement in EIBF by 44% (RR = 1.44, 95% CI = 1.06–1.97; one study; 390 participants; *QoE* = *high*) (43). However, three SRs (42, 50, 54) had reported significant heterogeneity (*I*^2^ between 78 and 98%).

###### EBF:

**Figure 3:**
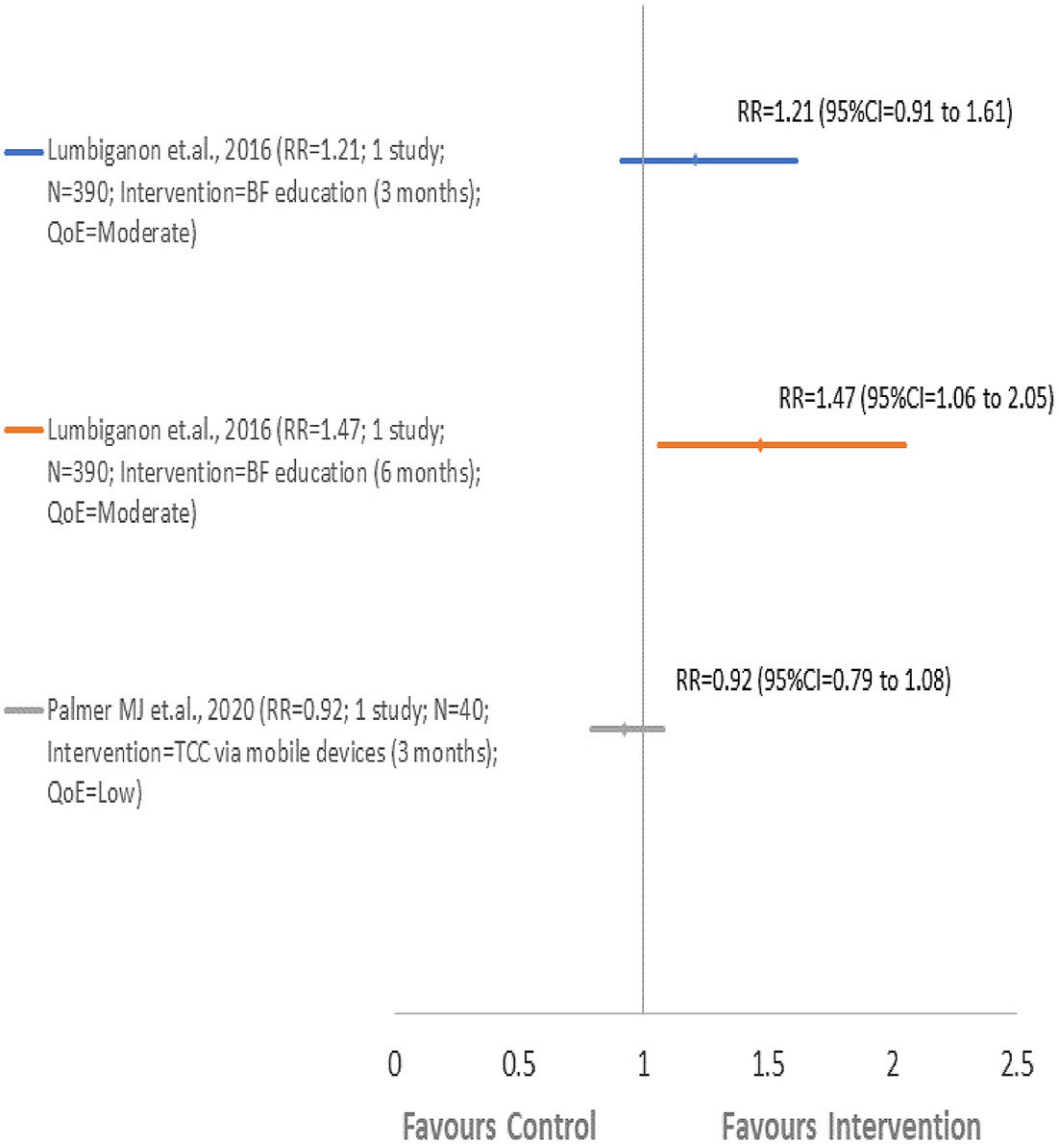
Comparison 1: BF intervention vs. routine care. EBF, exclusive breastfeeding.

Two included SRs (43, 55) reported EBF findings and concluded that monthly BF education sessions and weekly cell phone messages (43) reported the highest improvements in EBF at 6 months compared to TCC *via* mobile devices (55) (Figure 3; Table 2). An SR conducted by Lumbiganon et al. (43) assessed EBF at 3 and 6 months. Palmer et al. (55) evaluated EBF for up to 3 months in a low-risk setting (Kenya) where all women in the control group exclusively breastfeed. Therefore, the intervention did not have any impact on improving EBF as 100% of the women reported EBF.

Monthly BF education sessions and weekly cell phone messages delivered to women attending monthly microcredit meetings reported an improvement in EBF at 3 months by 21% (RR = 1.21, 95% CI = 0.91–1.61; one study; 390 participants; *QoE* = *moderate)* and at 6 months by 47% (RR = 1.47, 95% CI = 1.06–2.05; one study; 390 participants*; QoE* = *moderate)* (43). In another included SR, Targeted Client Communication *via* mobile devices delivered to pregnant and postpartum women and caregivers during the antenatal and postnatal period by health care workers reported a little improvement in EBF up to 3 months by only 8% (RR = 0.92, 95% CI = 0.79–1.08; one study; 40 participants; *QoE* = *low)* in low-risk setting (55). Significant heterogeneity, on the other hand, was not applicable.

##### Secondary outcome

No secondary outcomes such as Acceptability and Satisfaction were reported in this comparison group by any of the included SR.

#### Comparison 2: One BF intervention vs. other intervention

##### Primary outcome

###### EBF:

**Figure 4:**
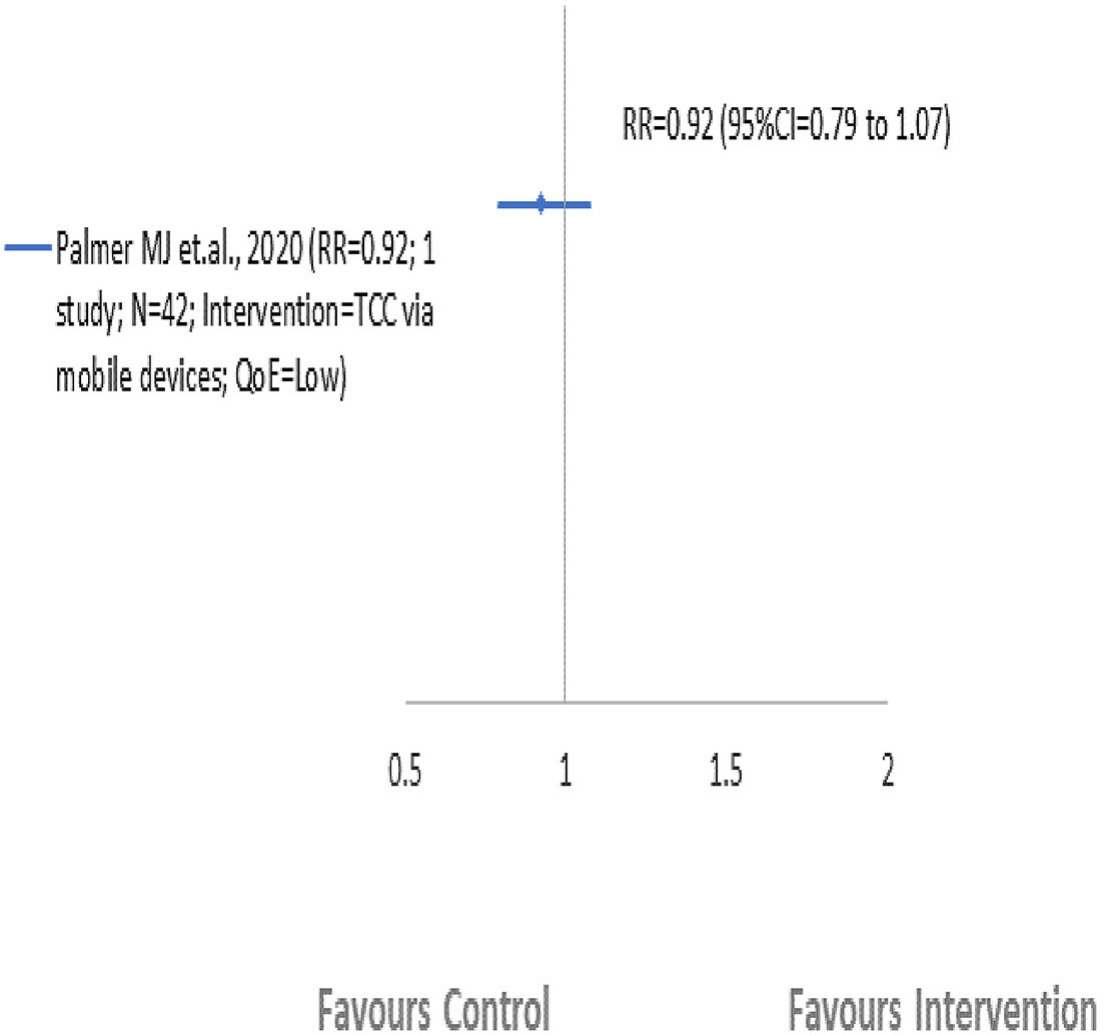
Comparison 2: One BF intervention vs. other intervention. EBF, exclusive breastfeeding.

Only one included SR (55) reported EBF findings (9 months postpartum) in this comparison group (Figure 4; Table 3). Palmer et al. (55) assessed that Targeted client communication (TCC) *via* mobile devices compared to non-digital TCC (pamphlets) had less or no effect on the improvement of EBF. TCC *via* mobile devices delivered to pregnant and postpartum women and caregivers during both antenatal and postnatal periods by health care workers showed an improvement of only 8% (RR = 0.92, 95% CI = 0.79–1.07; one study; 42 participants; *QoE* = *low)* (55).

##### Secondary outcome

No secondary outcomes such as Acceptability and Satisfaction were reported in this comparison group by any of the included SR.

## Discussion and recommendations for research and clinical practice

We aimed to identify specific interventions with the potential to promote and optimize breastfeeding practices. Our discussion had focussed on where we have found high-quality evidence of important effects. We listed key Cochrane reviews in need of an update. Finally, we had made recommendations for future systematic reviews and clinical research.

We had foreseen that there can be variations in the population, interventions, and outcomes of interest that can lead to heterogeneity. We also anticipated that there would be different approaches across SRs, across different author teams.

To reduce bias in the overview process, we followed standard review methods such as methods regarding duplication of effort, discussion-based resolution, and exclusion of overview authors from assessing their systematic reviews or trials. The inclusion of subject experts, public health experts, methodological experts, and information specialists strengthens the overview.

We intended to look into the evidence generated in LICs and LMICs and hence generalizability of the findings was restricted to LICs and LMICs. We evaluated six SRs that assessed the effects of different BF interventions to promote and optimize BF practices such as EIBF, EBF, and continued BF up to 2 years. The highest number of SRs addressed only two categories of primary outcome: EIBF and EBF. None of the SRs reported other outcomes of this overview such as continued BF up to 2 years, acceptability, and satisfaction.

In this overview, we used the R-AMSTAR tool for assessing methodological quality. Overall, the methodological quality of all the included SRs was high except for one (49) as it did not meet the R-AMSTAR tool criteria. The quality of evidence (QoE) reported in the included SRs varied from high to low.

The overview identified extensive evidence based on the type of interventions: educational intervention or support intervention, who delivered the interventions: digital or non-digital devices, personals from government, non-governmental, and private organizations, health system or health workers, non-healthcare professionals (women's group, peer counselors), and female ancillary nurse midwives (ANMs), recipients of the intervention: pregnant women, mothers of neonates, women in their reproductive age, mothers in their postpartum period, partners/spouses, or family members and the duration of the intervention: antenatal or postnatal phase.

Only two studies from LMICs (Nigeria and Iran) were included by Lumbiganon et al. (43). However, since there was no subgroup analysis for any of the listed outcomes, we did not extract the data from Iran and only retrieved the data from Nigeria. In another included SR, conducted by Palmer et al. (55), BF intervention did not show any significant improvements in BF practices since 100% of women in the control group reported EBF, and also the reported evidence was of very low certainty. Balogun et al. (42) included three studies from LMICs (Ghana, Nicaragua, and Nigeria) and one from LIC (Malawi). The study from Malawi, Nigeria, and Ghana addressed the impact of non-healthcare professional-led BF education on EIBF. However, a study from Nicaragua evaluated the impact of early mother-infant contact. In addition, the number of participants from Ghana was not available. Therefore, only two studies (Malawi and Nigeria) reported the data on EIBF.

In summary, all forms of included BF interventions were found to be effective in improving BF practices in many low-middle-income countries except for TCC *via* mobile devices which favors standard care and non-digital TCC since all of the women in the comparison group were exclusively breastfed their babies (55). The results were evaluated using two major comparisons groups: BF intervention against routine care and one BF intervention vs. other intervention comparison groups. Four SRs (42, 43, 50, 54) assessed the effect of interventions on EIBF whereas two SRs (43, 55) evaluated the effect of interventions on EBF in BF intervention vs. routine care comparison group. Only one included SR (55) reported EBF in another group i.e., one BF intervention vs. other intervention comparison group. However, no significant improvement was reported since the evidence was of very low certainty.

In the BF intervention vs. routine care comparison group, Community Based Intervention Packages (CBIP) delivered to pregnant and reproductive-age women during their ANC and/or PNC periods by Ancillary Nurse-Midwives reported the highest improvement in EIBF. However, insufficient evidence was reported to suggest that BF intervention showed improvements in EBF in both the comparison groups.

### Possible limitations, strength, and generalizability of the overview

#### Limitations of the review

This overview examined the available evidence concerning the interventions addressed to improve BF practices with no restrictions on the type of interventions. We did, however, limit our search to Cochrane reviews alone. We found limited reviews on the proposed topic. We acknowledge that not all SRs included in this overview came from LMICs or LICs only. We discovered that some of the SRs included participants ranging from low to high-income countries. We excluded some of the SRs that lacked participant subgroup analysis, even though they were designed to promote BF practices.

#### Strength of the review

The possible bias in the overview is estimated to be low. We followed the methods described in the Cochrane handbook (72). The search was as comprehensive as possible. Two authors independently screened studies, extracted data, and evaluated the methodological quality of reviews. We intended to investigate the evidence generated in LICs and LMICs and hence generalizability of the findings was limited to LICs and LMICs.

After an extensive literature search, we did not come across any overview of SRs that has addressed this area. Although, we found few SRs on interventions to promote BF practices while searching in Pubmed (32, 36, 73–77).

## Author's conclusion

### Implications for practice

BF interventions mentioned in this overview such as BF education sessions and support interventions, Community Based Intervention Packages, Community Health Educational Intervention delivered by health and non-healthcare professionals reported some improvements in the initiation of breastfeeding whereas insufficient evidence was reported for the improvement of EBF in LICs and LMICs. Among the different types of BF interventions, Community Based Intervention Packages (CBIP) was found to be most effective in improving BF initiation rates whereas BF intervention using multimedia such as TCC *via* mobile devices was found to be the least effective in promoting BF practices particularly in low and low-middle income countries.

This overview can assist personnel from government, non-governmental, and private organizations as well as a health system or health workers in raising awareness and encouraging pregnant women, mothers of neonates, women in their reproductive age, mothers in their postpartum period, partners/ spouses, or family members to improve BF practices.

### Implications of research

Based on the data extracted from included SRs, this overview highlights the gaps in primary research regarding the uncertainty about the settings such as LICs or LMICs, lack of evidence from LMICs, and also identified gaps in the availability of reliable up-to-date SRs on the effects of several BF interventions to promote and optimize BF practices. None of the included SR reported one of the primary outcomes i.e., continued BF up to 2 years of age and the duration of BF practices (Tables 2, 3). In addition, the included SRs did not evaluate any of the secondary outcomes such as acceptability and satisfaction (Tables 2, 3).

These identified gaps open up a new area of research and can be done on a priority basis. With an improved understanding of the significance of BF and the escalating risk of neonatal mortality and malnutrition due to inappropriate BF practices, there is an urgent need to perform good quality primary research on the mentioned interventions, especially in low-income (LIC) and low-middle-income countries (LMICs).

## Data availability statement

The original contributions presented in the study are included in the article/supplementary material, further inquiries can be directed to the corresponding author.

## Author contributions

AG proposed the concept of the review. MK developed and implemented the search strategies. AG and MK developed the protocol. SU and SQ screened the title and abstract. SS and PS screened full texts articles. Through conversation, AG resolved discrepancies for screening among primary reviewers. SU extracted data. AG and DS evaluated the methodological quality of included SRs. MK and SU drafted the manuscript with inputs from ST, AG, and SQ. All authors made significant contributions to this overview in reading, writing, and revision of the manuscript. All authors contributed to the article and approved the submitted version.
